# *Letter to the Editor:* Therapeutic Cooling Needs to be Faster and Deeper to Improve Outcomes!

**DOI:** 10.1089/ther.2021.0037

**Published:** 2022-06-07

**Authors:** Robert B. Schock, Douglas Kupas, Robert J. Freedman

**Affiliations:** ^1^ Life Recovery Systems, Kinnelon, New Jersey, USA.; ^2^ Department of Emergency Medicine, Geisinger Medical Center, Danville, Pennsylvania, USA.

**To the Editor:**

Granfeldt *et al.* (2021) and others have published studies that have challenged the guidelines for postcardiac arrest therapeutic hypothermia (TH). We have conducted our own review of studies in this field and conclude that TH best improves outcomes when provided sooner, faster, and to a truly therapeutic temperature. Specifically,

- cooling patients to <34°C is critical for making TH effective;
- it is crucial to cool patients as quickly and soon as possible after the ischemic event; and
- the use of temperature targets >34°C or cooling too slowly shows no benefit and may be deleterious.

We define rapid TH as reaching a core temperature of 32–34°C by cooling >3°C/hour while assuring the patient achieves TH within 3.5 hours of ischemic insult. Slower cooling methods leave the patient in the “shiver zone” (35.5–33.5°C) for hours, generating physiological stress and lactic acid in an already critically ill patient. Rapid cooling reduces this stressful time to minutes.

Five years ago this journal published a meta-analysis of 4700 postcardiac arrest patients treated with TH, which found that those treated with rapid TH had better outcomes compared with those treated with slower cooling (Schock *et al.*, 2016). Shockable rhythm patients rapidly cooled (using convective immersion surface cooling) reached 32–34°C in 40 minutes with 81% achieving good recovery (cerebral performance categories = 1 or 2), whereas only 57% of patients had good recoveries with slower cooling.

Kaneko *et al.* (2015), in a 467-patient analysis, observed that 88% of patients had good outcomes when resuscitated ≤30 minutes postcollapse and then rapidly cooled to 32–33.5°C (within 3.2 hours of resuscitation). Those cooled to 34–35°C had a 24% lower rate of good outcomes (*p* = 0.007).

“TTM” (targeted temperature management) includes both TH and controlled maintenance of normothermia. The TTM (Nielsen *et al.*, 2013) and TTM2 (Dankiewicz *et al.*, 2021) studies showed no improvements in outcomes of patients cooled to 33–34°C versus those maintained at 36–37°C. The TTM2 target of ≤34°C was reached ∼5 hours after resuscitation, and in the TTM study 33°C was not reached until 8 hours! The TTM trials cooled patients of very different demographics from the average U.S. out-of-hospital cardiac arrest patient; TTM trial subjects had much faster and higher rates of bystander cardiopulmonary resuscitation, and a much higher proportion of shockable rhythms than typically encountered by U.S. emergency medical systems. The TTM trials likely enrolled many patients whose brain injuries were so slight that TH was not required.

The analysis by Granfeldt *et al.* supports our conclusions that the use of TH target temperatures >34°C, cooling at rates <3°C/hour, or reaching target >3.5 hours after ischemic insult do not provide the full benefits of TH and fail to consistently improve recoveries. Rapid TH should be further considered to improve outcomes.
